# Sustained proton pump inhibitor deprescribing among dyspeptic patients in general practice: a return to self-management through a programme of education and alginate rescue therapy. A prospective interventional study

**DOI:** 10.3399/bjgpopen19X101651

**Published:** 2019-07-10

**Authors:** Cathal Coyle, Russell Symonds, Jane Allan, Sarah Dawson, Sheldon Russell, Adam Smith, Colin Daff, Helen Kotze

**Affiliations:** 1 Global Medical Affairs Lead, Global Medical Affairs, Reckitt Benckiser, Slough, Berkshire, UK; 2 NHS Business Manager, NHS team, Reckitt Benckiser, Slough, UK; 3 Nurse Advisor, Ashfield: Patient Solutions, Ashfield Healthcare, Leicester, UK; 4 Nurse Advisor, Ashfield: Patient Solutions, Ashfield Healthcare, Leicester, UK; 5 Practice Support Pharmacist, Medicines Management, Barnet Clinical Commissioning Group, London, UK; 6 Evidence and Outcomes Manager, Health Outcomes, Evidence Generation & Clinical Research, Reckitt Benckiser, Slough, UK; 7 Assistant Director, Medicines Management, Barnet Clinical Commissioning Group, London, UK; 8 Global Medical Affairs Manager, Global Medical Affairs, Reckitt Benckiser, Slough, UK

**Keywords:** alginates, proton pump inhibitor, dyspepsia, primary health care, self-care

## Abstract

**Background:**

Dyspepsia guidelines recommend that patients treated with proton pump inhibitors (PPIs) should step down to the lowest effective dose or return to self-care, but rebound hyperacidity can make this difficult. Many patients continue on PPIs in the long term, which may lead to safety and financial implications.

**Aim:**

To determine if a nurse-led educational support programme and rescue therapy for rebound symptoms can help patients achieve a sustained reduction in PPI use.

**Design & setting:**

A prospective interventional study was conducted at 26 surgeries across the UK.

**Method:**

Adult patients, treated with PPIs for ≥2 consecutive months with an active repeat prescription, were invited to a 20-minute dyspepsia clinic appointment with a trained nurse adviser. An action plan to reduce and/or stop their PPI usage was agreed and alginate supplied for the self-management of rebound symptoms. After 12 months, PPI status was reviewed and prescribing cost savings calculated.

**Results:**

After 12 months, 75.1% of 6249 eligible patients stepped down or off PPIs (35.3% stepped off; 5.0% stepped down then off; 34.8% stepped down only), while 8.7% of patients had reverted to their original PPI dose. PPI prescriptions fell from 89 915 to 45 880 and alginate prescriptions increased from 2405 to 6670. An average of 1.7 bottles (500 ml each) of alginate were used per patient who stepped down or off. Estimated annual cost-saving on prescriptions was £31 716.30.

**Conclusion:**

A programme of education and short-term rebound symptom management helped the majority of patients to successfully step down or off PPIs, significantly reducing the potential risks associated with chronic therapy.

## How this fits in

For many patients with dyspepsia on long-term PPI treatment, the potential for harm may be greater than the therapeutic advantage. However, symptoms related to rebound acid hypersecretion may represent a barrier to successfully reducing or stopping PPI treatment. A nurse-led programme of education and alginate prescribing for short-term rebound symptom management achieved sustained reductions in PPI use in 26 surgeries across the UK. This relatively simple intervention may significantly impact the risk of long-term harm to PPI-treated patients and empower them to take a more active role in their own care.

## Introduction

Since their clinical introduction during the 1980s, PPIs have become one of the most frequently prescribed drugs worldwide, with especially high rates of use in some European countries.^1^ As potent inhibitors of gastric acid secretion, they have revolutionised the treatment of acid-related conditions, including peptic ulceration^2^ and oesophagitis related to gastro-oesophageal reflux disease (GORD).^3^

While the short-term risk-benefit profile for PPIs is favourable, the impact of their widespread, long-term use is increasingly uncertain,^4–7^ with national drug agencies warning about their use long term and/or at high doses.^8,9^ Potential adverse effects could have a significant negative impact at a population level.^10^ Indeed, a recent large-scale study that followed PPI versus non-PPI users for a median of 5.71 years showed a significant association between PPI use and all-cause mortality, and the risk increased with prolonged use.^10^ Potential confounders associated with observational studies means results should be interpreted with caution, but the body of evidence does suggest the need to limit long-term PPI use to justified medical instances. For example, in patients with Barrett’s oesophagus, high-dose PPI use has been shown to significantly improve outcomes over a 9-year period.^11^

The UK guidelines for the management of dyspepsia and GORD recommend that patients receiving long-term PPI treatment are regularly reviewed and encouraged to step down to the lowest effective PPI dose, or return to self-care with antacids and/or alginates to manage intermittent symptoms.^12^ Despite this, a recent study reported that 60% of long-term PPI users in the UK had not attempted to discontinue or step down PPI dose^13^ and *Health Survey for England* revealed that PPIs were the third most commonly prescribed medicine, accounting for 11% of prescribed medicines in 2016.^14^ Evidence suggests that patients frequently persist on PPIs unnecessarily, and that PPIs are often prescribed without a clear indication.^15–21^ Furthermore, PPI treatment is often continued in GORD patients, even those with breakthrough symptoms and unsatisfactory symptom control.^22–24^

Action is required to support deprescribing and reduce the potential safety and financial implications of long-term PPI treatment. In practice, this can be challenging as sustained hypoacidity during treatment promotes hypergastrinaemia, causing rebound acid hypersecretion when PPI therapy is withdrawn.^25,26^ Stopping PPI treatment has been shown to induce acid-related symptoms after 4–8 weeks of treatment, even in previously asymptomatic individuals.^27,28^ While guidelines recommend restricting and deprescribing PPIs, they do not describe evidence-based strategies for the management of rebound hyperacidity. Thus, potential recurrence of symptoms may be a barrier to PPI reduction, especially in patients who have responded well to therapy.

An increasing body of evidence from small-scale, single-centre studies suggests that approaches using alginate as short-term rescue therapy can effectively help patients manage rebound symptoms and return to self-care.^29–33^ However, the effectiveness of this approach requires confirmation in a large, multi-centre study that allows for socioeconomic and cultural diversity. The aim of this study was to evaluate the efficacy and financial impact of a nurse-led programme of education and support across different regions of the UK.

## Method

### Study design and setting

**Figure 1.**
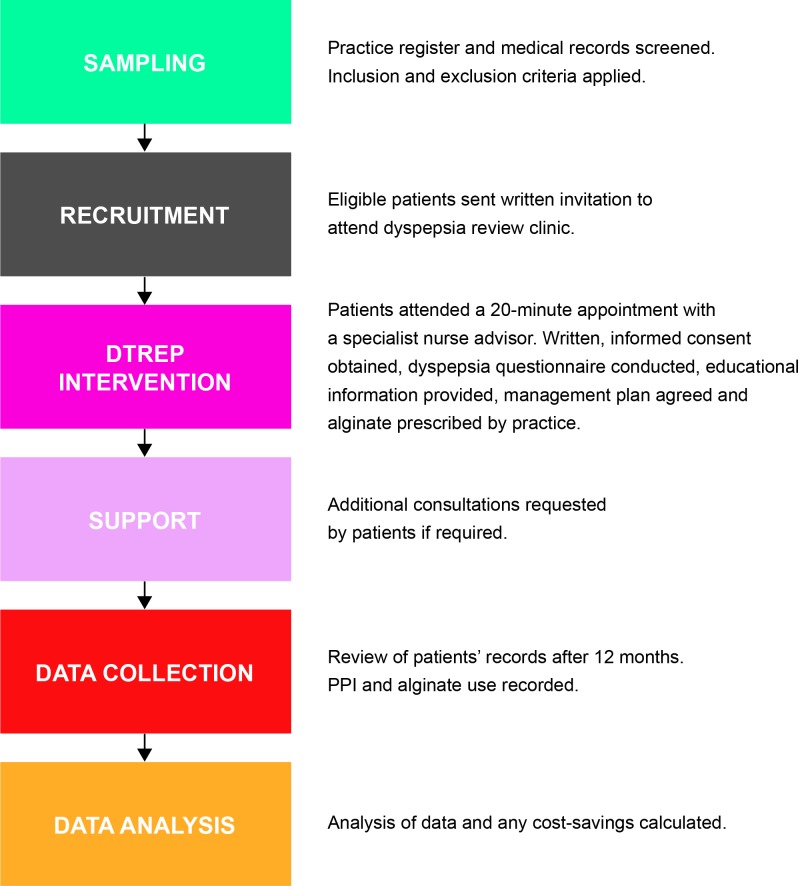
Study protocol summary DTREP = Dyspepsia Therapy Review and Education Programme. PPI = proton pump inhibitor.

This was a prospective interventional study conducted at 26 surgeries across nine regional primary care organisations (PCOs) in the UK (six in England, two in Scotland, one in Wales). Eligible PPI-treated patients participated in the nurse-led Dyspepsia Therapy Review and Education Programme (DTREP). In line with best practice guidance, DTREP aims to encourage patients on long-term PPI therapy to return to self-care through a package of education and support. Informed, written consent was obtained from all patients before entering the programme. An overview of the study methodology is provided in Figure 1.

### Patients

The electronic register for each surgery was searched to identify all potentially suitable patients. Eligible patients (aged 18–90 years) had an active repeat prescription for PPIs and had been treated with PPIs for a minimum of 2 consecutive months. Specialist nurse advisers screened medical records, applying pre-specified inclusion and exclusion criteria, before providing GPs with participant lists for approval.

Exclusion criteria included patients on *Helicobacter pylori* eradication therapy; with grade 3 or 4 oesophagitis, Zollinger-Ellison syndrome, terminal illness, history of oesophageal varices, strictures, or dilation; awaiting or under gastrointestinal clinic or gastroscopy review; receiving immunosuppressants, chemotherapy, or radiotherapy; or with alarm symptoms (persistent vomiting, bleeding, anaemia, unexplained weight loss, or difficulty swallowing).

Patients who were considered for step down to the lowest maintenance dose of PPI but could not proceed to self-management included those with a history of peptic ulceration associated with *C* *ampylobacter*-like organism negative status, a Barrett’s oesophagus diagnosis, or patients requiring continuation of non-steroidal anti-inflammatory drug (NSAID) therapy, except for those considered at high risk; that is, those with previous ulceration, those on other medication harmful to the gastric and duodenal lining, older people, and those on long-term high-dose NSAIDs (20 mg omeprazole defined as maintenance dose for NSAID coverage). Patients using aspirin or clopidogrel to prevent cardiovascular disease could be stepped off PPIs, except if considered high risk; for example, previous ulceration, taking medication harmful to the gastric/duodenal lining, and older people.

### Dyspepsia therapy review and education programme

All eligible patients were sent written invitations to attend a 20-minute dyspepsia clinic appointment with a specially trained nurse adviser. During the initial visit, patients completed a patient counselling questionnaire (further information available from the authors on request) to obtain a structured history and to screen for alarm symptoms. Symptomatic patients who had not undergone screening for *H. pylori* infection may have been referred to the practice nurse for testing according to the local guidelines and were subsequently re-entered into the programme on completion of eradication therapy. As per UK National Institute for Health and Care Excellence guidelines,^12^ patients were given verbal and written educational information about their condition, its causes, risk factors, alternative treatment options, and lifestyle factors. This included advice about identifying personal triggers and potential dietary precipitants such as coffee, chocolate, and fatty foods.^34^ Risk factor management included a brief alcohol intervention and smoking cessation referral advice, if relevant. The specialist nurse adviser and the patient agreed a specific action plan to reduce and/or stop PPI usage. As part of this plan participants were prescribed, as per GP practice guidance, Gaviscon Advance, an alginate formulation (1 g sodium alginate and 200 mg potassium bicarbonate/10 ml dose) licensed for rebound dyspepsia and breakthrough symptoms. Further appointments were offered to all patients according to their individual needs. Patients who had consented to participate but did not attend the clinic were sent a further invitation letter offering flexible appointment times. Specialist nurse advisers reviewed patients’ records of PPI and alginate prescription for the 12-month period following intervention, and again at 24 months at several surgeries. Any adverse events were recorded using recognised protocols.

### Data collection and analysis

All data were coded and collated in a Microsoft Excel workbook (version 6.4). Simple descriptive statistics were used to calculate the number and percentages of patients who had stepped down (reduced PPI dose) or stepped off (discontinued) PPIs after 12 months.

The net financial costs of PPI and alginate use were calculated after 12 months. All annualised cost estimates were derived from the UK Department of Health and Social Services electronic drug tariff. Annual savings were calculated by comparing PPI costs at baseline with PPI and Gaviscon Advance costs at study conclusion.

## Results

### Patients

Prescribing data were reviewed for 231 900 patients; 14 108 PPI-treated patients were identified, of whom 6249 were eligible for this study.

### Outcomes

#### One-year review

After participating in the DTREP, a total of 4691 (75.1%) eligible patients had stepped down or off PPIs after 12 months (Table 1). PPIs were discontinued in 40.3% of eligible patients. Overall, around a third of patients stepped off (35.3%), another third stepped down to a lower dose of PPI without stepping off (34.8%), and 8.7% of patients reverted to their original PPI dose after 12 months.

**Table 1.** Net impact of DTREP programme on proton pump inhibitor status

Outcome	Patients, *n* (%)
Total eligible	6249 (100)
**Step down** **Step down then off** **Step off only**	2173 (34.8)312 (5.0)2206 (35.3)
**Total successful reduction**	4691 (75.1)
**Did not attend** **Unsuccessful, reverted** **Under review and/or refused** **No longer active**	520 (8.3)541 (8.7)163 (2.6)334 (5.3)
**Total reduction**	1558 (24.9)

DTREP = Dyspepsia Therapy Review and Education Programme.

**Figure 2.**
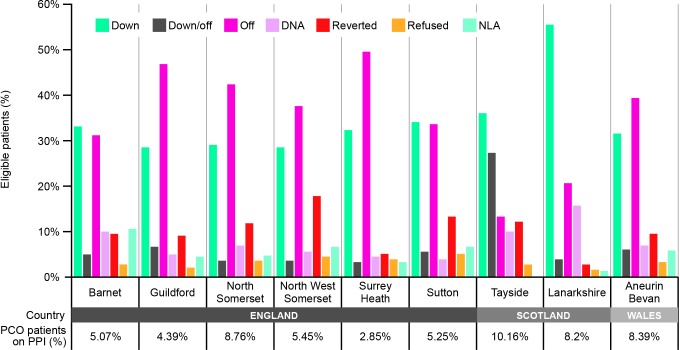
DTREP impact on PPI use across regional PCOs in England, Scotland, and Wales The proportion of PPI-treated patients and change in PPI status are summarised for each study PCO. DNA = did not attend. DTREP = Dyspepsia Therapy Review and Education Programme. NLA = no longer active. PCO = primary care organisation (clinical commissioning group). PPI = proton pump inhibitor.

In England and Wales (Figure 2), the rate of stepping off PPIs (England 43.8%, Wales 44.6%) was greater than stepping down (England 30.2%, Wales 31.0%), whereas data from the Scottish PCOs show greater rates of stepping down (52.6%) versus stepping off (26.1%). Rates of non-attendance were also noticeably higher in Scotland (14.7%) compared with England (6.8%) and Wales (6.7%). No adverse events were reported during the study period.

#### Two-year follow up

Three of the study surgeries in England also reviewed prescribing data 24 months after implementation of DTREP. Out of 1455 PPI-treated patients, 669 were eligible. Two years after they entered DTREP, 254 (38.0%) had stepped off PPIs and 174 (26.0%) had stepped down. Nine out of the 254 (3.5%) patients who stepped off had first stepped down.

After 24 months, 64.0% of patients had either stepped down or off PPIs, 14.2% were unsuccessful and/or reverted, 7.9% did not attend or were under review and/or refused to complete the programme, and 13.9% were no longer active (that is, no longer registered with the surgery).

#### Prescriptions and costings

**Figure 3.**
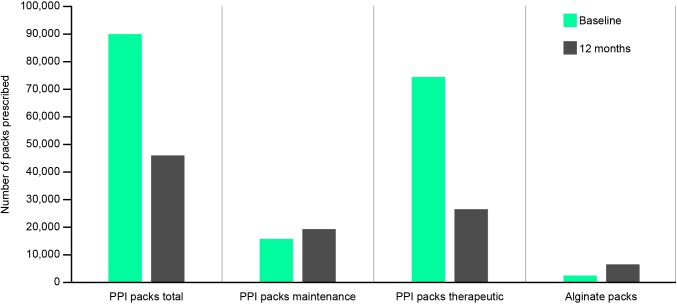
Net impact of DTREP programme on prescriptions DTREP = Dyspepsia Therapy Review and Education Programme. PPI = proton pump inhibitor.

Overall, there was a 49.0% reduction in PPI prescribing over the 1-year review period (Figure 3), with the number of PPI prescriptions falling from 89 915 to 45 880. The number of alginate prescriptions over the same period increased 2.7-fold, from 2405 to 6670. During the 12-month period, an average of 1.7 bottles (500 ml each) of Gaviscon Advance were used per patient who stepped down and/or off. Taking into consideration the cost of alginate (£21 836.80), the net prescribing cost-saving from DTREP was £31 716.30 per annum.

## Discussion

### Summary

Provision of education and support through the nurse-led DTREP was very effective at helping patients successfully reduce their PPI use across all the study PCOs. Overall, around 40% of patients stopped PPI treatment completely and 35% reduced their dose. These changes in PPI use were achieved without any reported safety concerns and were maintained long term; PPI reduction was maintained in 75.1% and 64.0% of patients at 12 and 24 months, respectively.

An important element of DTREP was preparing patients for the possible recurrence of symptoms and provision of alginate as rescue therapy. Gaviscon Advance is a concentrated alginate formulation that forms a resilient reflux-suppressing raft at the gastro-oesophageal junction.^35,36^ Its rapid action makes it an ideal candidate for rescue therapy and it has been shown previously to help PPI-treated patients cope with acute breakthrough symptoms.^37^ Furthermore, the non-systemic, physical mode of action means it has a favourable safety profile with no drug interaction issues.^38,39^ During the 12-month study period, an average of 1.7 bottles of alginate were used for every patient who stepped down and/or off, suggesting that a real reduction in medication use was achieved rather than simply switching one chronic medication for another. Furthermore, implementation of DTREP was estimated to have saved more than £30 000 on the annual prescribing budget across the study surgeries.

### Strengths and limitations

A notable strength of this study was the large patient sample (more than 6000 eligible PPI-treated patients), real-world setting, and regional spread of the study population, which represents diverse social, economic, and cultural groups. The overall effectiveness across the UK demonstrates the widespread feasibility of DTREP for general clinical practice. A limitation of the study was that patients were not well-characterised in terms of age, duration of PPI use, ethnic group, and health and socioeconomic status — factors that will almost certainly influence the likelihood of successful PPI reduction. The proportion of PPI-treated patients within the individual PCOs may give an indication of the general health status of the local population. The PCO with the highest proportion of PPI-treated patients (Tayside, 10.2%) had a considerably greater rate of ‘step down then off’ versus ‘step off alone’ (26.9% versus 13.1%, respectively). This contrasts with the PCO with the lowest proportion of PPI-treated patients (Surrey Heath, 2.9%), where patients were more likely to immediately step off (49.1%), rather than step down first (1.6%). Rates of non-attendance were also generally higher in the PCOs with a greater proportion of PPI-treated patients. Further investigation into how cultural, health, and socioeconomic factors influence the likelihood of step down versus step off will be important in helping to adapt DTREP to better meet the needs of the local population. Future studies should also assess the health-related quality of life benefit for patients versus the cost of the programme.

A further limitation is that, with no comparator, the relative contribution of alginate is unknown. Also, use of over-the-counter alginate or other medication for dyspepsia was not recorded, the costs of running the programme were not taken into account in the estimated overall cost-saving calculations, and there was no guarantee of consistency in approach between centres.

### Comparison with existing literature

As mentioned, the relative contribution of alginate to the success of DTREP cannot be confirmed. However, previous studies using educational intervention alone had limited or no success in reducing PPI use.^19,40,41^ The data in this study are also consistent with previous small-scale studies involving similar interventions of educational support and alginate for rebound symptom management.^29–33^ One of these studies reviewed PPI use as part of a polypharmacy medicine optimisation review (PMOR).^33,42^ It revealed that 79% of patients using PPIs were taking four or more medications, and PMOR not only led to PPI reduction but also reduced NSAID and selective serotonin reuptake inhibitor use. This suggests that similar interventions could be applied to other areas of medicine where patients might benefit from reducing their long-term medication. Furthermore, in addition to reduced PPI prescribing, PMOR led to an unexpected decrease in gastrointestinal and endoscopy referrals, indicating a potential for cost-savings owing to reduced healthcare utilisation.

### Implications for practice

PPI overprescribing has been a recognised problem for more than a decade,^43^ but the number of PPI items dispensed in the UK continues to rise, doubling from 29 million in 2007 to 59 million in 2017.^44^ A recent study investigating PPI prescribing in primary care found that up to 21% of PPI courses had no coded indication.^13^ Unnecessary PPI use puts patients at risk of side effects, such as small intestine bacterial overgrowth,^45^ or long-term complications, such as increased fracture risk.^46^ While rebound symptoms^27^ make it difficult for patients to reduce their PPI dose or return to self-management in line with guidelines,^12^ the success of DTREP emphasises that a relatively simple intervention can help patients achieve this. The results of DTREP are a reminder that high-volume prescribing can be successfully reduced, and are particularly encouraging in light of the recent focus on empowering patients with evidence-based approaches to self-care.^47^

A population modelling tool used DTREP data to forecast outcomes at a population level, taking into account prescribing costs and drug-related complications based on national averages. The tool shows that for 100 000 registered patients, 6.1% of whom are taking PPIs, 937 patients could be stepped down or off, with a net cost-saving of £13 676.71 (based on costings as of 1 August 2018).

Three out of four patients who entered the DTREP achieved a sustained reduction or complete discontinuation of PPIs using alginate as a short-term rescue therapy.

Greater guidance on rebound symptom management and broader use of DTREP has the potential to significantly impact the risk of long-term harm to PPI-treated individuals. Such interventions of education and support will help drive implementation of evidence-based guidance and empower patients to take a more active role in their own health care.
